# Racial/Ethnic Differences in Arrest Probability Trajectories after Traumatic Brain Injury: A Model Systems Study

**DOI:** 10.3390/healthcare10102078

**Published:** 2022-10-19

**Authors:** Mickeal Pugh, Paul B. Perrin, Juan Carlos Arango-Lasprilla, Daniel W. Klyce, Shawn C. T. Jones, Natalie D. Dautovich

**Affiliations:** 1 Department of Psychology, Virginia Commonwealth University, Richmond, VA 23284, USA; 2 Department of Psychology, School of Data Science, University of Virginia, Charlottesville, VA 22904, USA; 3 Polytrauma Rehabilitation Center TBI Model Systems, Central Virginia Veterans Affairs Health Care System, Richmond, VA 23249, USA; 4 Department of Physical Medicine and Rehabilitation, Virginia Commonwealth University, Richmond, VA 23298, USA; 5 Traumatic Brain Injury Unit, Sheltering Arms Institute, Virginia Commonwealth University, Richmond, VA 23233, USA

**Keywords:** traumatic brain injury, rehabilitation, racial/ethnic disparities, arrests, TBI model systems

## Abstract

**Background:** Previous literature has documented racial/ethnic differences in traumatic brain injury (TBI) risk, cause, treatment, and rehabilitation. The purpose of the current study was to investigate potential racial/ethnic differences in arrest probability trajectories over the first 10 years after TBI and whether injury and sociodemographic characteristics accounted for these differences. **Methods:** The current study included 13,195 participants with moderate-to-severe TBI in the TBI Model Systems National Database who had arrest data from at least one follow-up time point (Years 1, 2, 5, and/or 10). A series of hierarchical linear models assessed racial/ethnic differences in trajectories of arrest probability over these 10 years post-injury and then included socio-demographic and injury-related covariates. **Results:** White individuals with TBI had lower arrest probability trajectories than Black and Native American individuals, and Asian individuals with TBI had lower arrest probability trajectories than White, Black, Latinx, and Native American persons. In many cases, racial/ethnic disparities persisted even when injury and sociodemographic characteristics were covaried. **Conclusion:** These results suggest that rehabilitation clinicians should assess for post-injury arrest risk factors such as age, sex, education, pre-injury unemployment, arrest history, and substance abuse, particularly in Black, Latinx, and Native American groups, and integrate programming to lessen post-injury arrest probability and improve overall rehabilitation outcomes.

## 1. Introduction

TBI rates grew between 1990 and 2016 from 3.6% to 8.4% global prevalence [1], with roughly 27 million new cases of TBI occurring in 2016 [2]. American Indians/Alaskan Natives have the highest rates of age-adjusted hospitalization for TBI, whereas Asians have the lowest [3] The incidence rates for TBI are much higher for Black individuals than White, with 485 per 100,000 compared to 399 per 100,000, respectively [4]. Black, Latinx, and Native American groups are more likely to have a violent cause of TBI, compared to White persons [5,6,7,8,9,10,11]. These racial/ethnic differences in TBI rates have also been shown to translate into respective differences in rehabilitation outcomes.

Racial/ethnic minority individuals with TBI show higher disability and functional limitations and unstable employment compared to White individuals [6,7,8,9,12]. Compared to White individuals, Latinx people with TBI have reported higher functional disability [9]. These post-injury differences exist even after controlling for pre-injury characteristics such as education, age, and gender, as well as cause of injury [8,13]. Research has also found higher depression levels among Black individuals with TBI [14] and a greater degree of symptom clusters similar to PTSD compared to White individuals [15].

Although racial/ethnic differences in functional outcomes following TBI have received research attention, the field has investigated criminal arrests as an event following TBI only minimally with no substantive work focusing on different rates by race/ethnicity. Vaughn and colleagues [16] presented prevalence rates of TBI among a sample of incarcerated persons, predictors of arrest history, and long-term correlates of recidivism. One-third of their sample screened positive for TBI, with approximately 64% mild, 12% moderate, and 12% severe. Vaughn and colleagues [16] conducted a survival analysis among a prison population predicting recidivism, and their results showed that more than half (53%) of the sample was rearrested between 12 and 30 months following release, and prior history of TBI, racial/ethnic minority status, and prior arrests contributed to this outcome. Male sex, younger age, and lower educational achievement have been associated with pre-injury arrest history among populations with TBI [17,18,19]. History of psychological treatment [17], antisocial personality disorder [18], major depression [20], and substance abuse [19,20] have been shown to predict criminal arrest outcomes after TBI. Importantly, violent cause of TBI, particularly assaults, were associated with pre-injury arrest history [17]. Black and Latinx individuals with TBI were more likely to have an arrest history compared to their counterparts [21]. Literature has shown violent injury cause was more common among Black, Latinx, and Native American individuals, compared to Whites [5,6,7,8,9,10,11]. Sociodemographic risks after TBI for criminal justice concerns are under-explored within Native American populations. However, literature has shown this community to experience harsher sentencing following criminal conviction compared to White, Black, and Latinx groups [22].

Only two studies to date have investigated possible racial/ethnic differences in arrest probability after TBI. Elbogen et al. [23] found that younger age, lower education, male sex, longer loss of consciousness, and intact motor functioning predicted higher arrest probability at 1, 2, and 5 years after TBI, but the authors did not find racial/ethnic differences. In their analyses, all racial/ethnic minority-identified individuals were placed into the same comparison group, but as noted above, different racial/ethnic minority groups experience vastly different rates of and outcomes from TBI (e.g., Native American vs. Asian). In the second study, Miles et al. [24] assessed these outcomes among a veteran population with TBI and found that moderate TBI, pre-injury arrest history, having received mental health treatment, moderate to heavy alcohol use, and having one more follow-up interview independently contributed to post-TBI arrests across a span of ten years. Similarly, this study did not find significant racial/ethnic differences in arrest outcomes when comparing Black vs. White, Hispanic vs. White, or “Other race/ethnicity” vs. White, although the veteran nature of the sample may have excluded a larger number of individuals who otherwise would have had pre-injury risk factors for criminal arrest (e.g., history of criminal arrest, serious psychiatric history, lack of a high school diploma, etc.).

No research to date has explored racial/ethnic differences in the trajectories of arrest probabilities among individuals with TBI. The current study included a large sample of participants with a history of moderate-to-severe TBI in the TBI Model Systems National Database who had arrest data from at least one follow-up time point over the first 10 years after TBI. It examined racial/ethnic differences in trajectories of arrest probability after TBI and included socio-demographic and injury-related covariates to investigate whether these variables explained any racial/ethnic differences found. Previous literature has identified racial/ethnic differences in violent cause of TBI, criminal arrests, and functional outcomes, as outlined above. Therefore, the current study hypothesized that Black, Latinx, and Native American persons with TBI would show higher arrest probability trajectories compared to White and Asian groups. Moreover, it was expected that racial/ethnic differences in criminal arrests following TBI would in part be accounted for by injury-related and sociodemographic variables.

## 2. Method

### 2.1. Participants

The current study included a sample of 13,195 participants who were enrolled in the National Database of the National Institute for Disability, Independent Living, and Rehabilitation Research (NIDILRR) TBI Model Systems (TBIMS). The TBIMS is comprised of individuals with newly acquired TBI who receive comprehensive inpatient rehabilitation services at one of the TBIMS sites in the U.S. Inclusion criteria for the TBIMS National Database are (a) age 16 years or older at the time of injury, (b) medically diagnosed TBI from the TBIMS center, (c) either Glasgow Coma Scale score of ≤12 upon emergency admission, >24 h duration of posttraumatic amnesia (PTA), loss of consciousness (LOC) >30 min, or evidence of intracranial trauma on neuroimaging (i.e., to indicate mild complicated, moderate, or severe TBI), (d) admission to the respective TBIMS acute care hospital within 72 h of injury, and (e) enrollment and completion of inpatient rehabilitation services within the TBIMS center. Participants were asked, “What racial group or groups do you most identify as?” They selected a single option among the following: White, Black, Asian/Pacific Islander, Native American, Hispanic origin (re-named in this document as “Latinx”), other, or unknown. The term “Latinx” and its acceptance continues to evolve over time, and it is meant to refer to people of Latin American origin and descent (Salinas Jr., 2020). This term, along with all other racial/ethnic group classifications, often assume homogeneity in each identity, overlooking within group cultural variability. Thus, for the current study, racial/ethnic group coding only refers to how participants self-reported their racial/ethnic identity out of the options given within the larger TBIMS data collection. Inclusion criteria for the current analyses were (a) reported race/ethnicity, (b) have preinjury arrest data, and (c) have arrest data for at least one of the follow-up time points of interest (1, 2, 5, and 10 years).

### 2.2. Measures

**Arrests.** At baseline data collection, participants or their surrogates reported pre-injury arrest information with a response to the prompt “Were you [or was the person with brain injury] ever incarcerated for conviction of a felony?”. At each follow-up interview, participants or their surrogates reported a history of arrest in the year prior to data collection by responding to the prompt “In the past year have you [or has the person with brain injury] been arrested?”. Both of these responses were dichotomized as 0 (no) and 1 (yes). The question that assesses pre-injury arrests requires several conditions, which are: (a) arrested prior to their injury, (b) conviction for felony, and (c) incarceration for that offense. Thus, pre-injury criminal activity reflects incarceration with a conviction for felony, as opposed to any arrest or convictions for lesser chargers, whereas post-injury criminal activity reflects any arrest in the past year, irrespective of the chargers or any subsequent conviction.

**Injury Characteristics.** TBIMS sites collect data on TBI features such as the cause (e.g., falls, motor-vehicle accident, assault, etc.) and severity of the injury (e.g., initial GCS, time to follow commands, and duration of PTA). The current study operationalized violent TBI as TBI caused by “gunshot wounds”, “assault with a blunt instrument”, or “other violence”, per the TBIMS classification system. Duration of PTA indicated injury severity for the current study, and Department of Defense/Veteran’s Affairs (VA/DoD, 2016) clinical practice guidelines for classification of TBI were used, as a PTA of 0 to 1 day indicated mild TBI, >1 day and <7 days moderate, and >7 days severe.

**Post-TBI Functional Independence**. The Functional Independence Measure (FIM; [25]) is an 18-item assessment that examines the functional status of persons with disabilities. Respondents rate each item on a 7-point scale (1 = complete dependence, 7 = complete independence), and total scores range from 18 to 126 with higher values reflecting greater independence. The FIM has strong internal consistency, particularly for TBI populations (*α* = 0.93; [25]) and this was consistent for the current study (*α* = 0.85). The FIM is rated by certified assessors at admission, discharge, and follow-up, although only the discharge score was used as a covariate.

**Substance Use.** TBIMS centers assess pre- and post-injury problematic substance use by (1) have taken illicit drugs, (2) in the past month have binge drunk alcohol (e.g., 5 or more drinks in one setting), or (3) their alcohol consumption was categorized as “Heavy” (e.g., 14 drinks per week for men and seven for women, based on the items adapted from the CDC’s risk Factor Surveillance System [26].

**Demographics**. Participants or their surrogates reported demographic data, including age (e.g., years), sex (e.g., male or female), race/ethnicity, years of education, and employment status. Race/ethnicity data were coded as White (1), Black (2), Asian/Pacific Islander (3), Native American (4), Hispanic origin (5), other (7), or unknown (9), based on 1990 and 2000 U.S. Census guidelines.

### 2.3. Procedure

After enrolling participants in the TBIMS, their health and social history were collected from medical records and participant/surrogate interviews. TBIMS research staff collected follow-up data at 1, 2, 5, and 10 years as close to the injury anniversary date as possible via telephone interviews, and participants could either choose an in-person interview or complete a self-administered questionnaire.

### 2.4. Data Analyses

**Preliminary Analyses.** The current study computed descriptive statistics (i.e., means, standard deviations, frequencies, and percentages) of demographic and injury-related characteristics broken down by race/ethnicity, assessing for statistically significant differences among racial/ethnic groups using analyses of variance (ANOVAs) or chi-squared analyses as appropriate. Normality tests (i.e., skewness and kurtosis) were conducted to determine whether the continuous scales and subscales were normally distributed and critical values of 2.0 were used to identify variables that were skewed or kurtotic. Data were checked for multicollinearity via correlation coefficients among all predictor variables (with a goal *r* < 70). Regarding missingness, Little’s Missing Completely At Random (MCAR) test assessed the degree to which these data were missing at random.

**Primary Analyses.** The main study analysis, hierarchical linear modeling (HLM), used full information maximum likelihood (FIML) estimation procedures without imputation to include participants with missing data. Several sets of HLMs were performed following standard preparation and interpretation guidelines established by Kwok et al. [27] with arrests during the prior year (1 = yes, 0 = no) at each follow-up period as the outcome. The study conducted a conditional (null) model to assess data clustering of arrest probability variance among study participants to evaluate whether to proceed with HLM. The unconditional growth model determined whether a linear (e.g., straight line), quadradic (e.g., U-shaped), or cubic (e.g., S-shaped) model most accurately reflected arrest probability over time. The next set of HLMs assessed differences in arrest probabilities over time among racial/ethnic groups. For each analysis, only participants who identified as the one of two racial/ethnic groups being compared (e.g., Black vs. White) were included—thus, all trajectory comparisons were pairwise. The next set of HLMs incorporated interaction terms between time and race/ethnicity, when racial/ethnic effects had previously been statistically significant, to determine if these differences in arrest probability occurred differentially as a function of time (i.e., a different slope of arrest probability trajectory over time as a function of race/ethnicity). The final set of HLMs introduced possible covariates (e.g., demographic, injury) supported by previous research to examine the effects of those covariates on racial/ethnic differences in arrest trajectories, as well as in differential racial/ethnic effects over time, where present. The covariates included were age, sex, preinjury arrest history, injury severity (e.g., duration of PTA), injury cause (e.g., violent versus non-violent), functional independence at discharge, and preinjury features (e.g., education, competitive employment, and problematic substance use). All analyses were conducted using SPSS 26.0 (IBM, New York, NY, United States) and two-tailed significance was established at an alpha of 0.05.

## 3. Results

### 3.1. Preliminary Analyses

Table 1 presents descriptive statistics (i.e., means, standard deviations, frequencies, and percentages) for sample demographics, psychosocial features, and injury characteristics by racial/ethnic group with statistically significant differences noted. Generally, White and Asian participants were the oldest subgroups at injury, and Black and Latinx individuals were the most likely to be male. Asian participants had the highest education level, although Latinx participants were the most likely to be employed, with Native Americans the least likely. Asian participants were the least likely to have a pre-injury substance use or arrest history, and Native Americans the most likely in both categories. Black individuals were the most likely to have had a violent cause of injury. Latinx individuals had a higher proportion of severe injury levels (categorized by PTA) relative to White and Asian individuals, and Black individuals with TBI had the lowest FIM scores at discharge. An analysis of arrest probability differences by racial/ethnic group independently at each time point showed Asian individuals with TBI generally had the lowest arrest probability at the first three time points. White individuals with TBI generally had lower arrest probabilities compared to Black and Native American individuals, and Native American individuals with TBI generally had higher arrest probabilities compared to Black and Latinx individuals with TBI.

The study created a correlation matrix to examine bivariate correlations among demographic and injury-related variables with arrest history at each time point (Table 2). The following coding scheme was used in both the correlation matrix, as well as in the successive HLMs: participant sex, female = 0 and male = 1; pre-injury employment, 0 = not competitively employed and 1 = competitively employed; pre-injury substance use, 0 = no and 1 = yes; pre-injury (felony) incarceration history, 0 = no and 1 = yes; injury severity, 0 = mild, 1 = moderate, and 2 = severe; injury cause, 0 = non-violent and 1 = violent; and post-injury arrest in prior year, 0 = no and 1 = yes). Results from the correlation matrix showed that male sex, younger age, being non-competitively employed at injury (aside from years 2 and 10), lower education, pre-injury substance use and arrest history, and violent injury cause and severity (aside from year 10) were associated with higher arrest probabilities over time.

Regarding normality of continuous predictor variables, skewness ranged from −0.14 to 0.63, and kurtosis ranged from −0.57 to 1.33; these values did not exceed the critical value of 2.0 and the analysis of multicollinearity did not exceed the critical value of *r* > 0.70 (Table 2). Regarding missingness, the percentage of missing data for arrest data were 9.7, 22.5, 45.8, and 70.4 at one, two, five, and ten years, respectively. Results of Little’s MCAR test were significant (*χ*^2^ 28 = 190.82, *p* < 0.001). This test indicated the data were not missing completely at random. As a result, we retained individuals with missing data with FIML instead of using listwise deletion for cases with missing data.

### 3.2. Identifying Curvature Models

The unconditional model yielded a statistically significant estimated participant variance of 0.011 (Wald *Z* = 26.45, *p* < 0.001), as well as a statically significant estimated residual variance of 0.044 (Wald *Z* = 100.54, *p* < 0.001). The intraclass correlation coefficient was calculated to be 0.20, indicating that approximately 20% of the total variance of arrest probability was associated with the participant grouping and that the assumption of independence was violated. This suggested there was appropriate clustering of arrest probability variance within participants to proceed with HLM.

Results of the unconditional growth (linear), quadratic (U-shaped), and cubic (S-shaped) models indicated that a quadratic trajectory (df = 6, −2LL = −3629.25) of arrest probability was the best fit compared to linear (df = 5, −2LL = −3623.78) and cubic (df = 7, −2LL = −3629.79) trends, given that the critical *χ^2^* difference exceeded 3.84. This pattern displayed a gradual decrease across one, two, five, and ten years with a very slight leveling off of the curve, contributing to the quadratic movement.

### 3.3. Racial/Ethnic Differences

**Figure 1:**
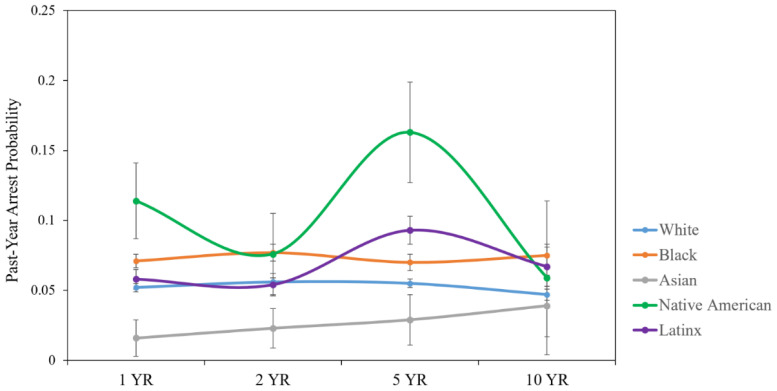
Racial/ethnic differences in past-year arrest probability trajectories over time with unadjusted means and standard error bars.

A second set of HLM analyses (ten total) examined whether significant differences in arrest probability over time were present between racial/ethnic groups. Race/ethnicity (dummy coded for two-group comparisons), quadratic time, and linear time were included in the models as fixed effects. All statistically significant and non-significant racial/ethnic differences analyzed in the ten HLMs, as well as their unstandardized *b*-weights and *p*-values, appear in Table 3. The arrest probabilities for each racial/ethnic group over time appear in Figure 1.

Relative to White individuals with TBI, Black and Native American individuals had elevated arrest probability trajectories over time. Asian persons with TBI had a lower arrest probability trajectory than White, Black, Latinx, and Native American individuals. Latinx individuals with TBI had a lower arrest probability trajectory than Native American individuals.

### 3.4. Differential Effects of Race/Ethnicity over Time

For the third set of HLMs, quadratic time * race/ethnicity interaction term was added, as well as the lower order interaction term (e.g., linear time * race/ethnicity) when a main effect of race/ethnicity had been present in one of the prior HLMs. The results appear in Table 4. This series of analyses found that for the previously significant main effect variables, there was no differential change in arrest probability trajectories over time as a function of race/ethnicity.

### 3.5. Accounting for Racial/Ethnic Differences

The next set of HLM analyses was performed for all statistically significant racial/ethnic trajectory comparisons originally found and introduced demographic and injury-related characteristics as possible covariates to examine whether they accounted for these racial/ethnic differences. In this set, linear time, quadratic time, age, sex, pre-injury arrest history, employment status, pre-injury substance use, education, injury severity, post-TBI functional independence, and violence as a cause of injury were added as covariates. All statistically significant and non-significant racial/ethnic differences in arrest outcomes over time after including demographic and injury-related characteristics as covariates appear in Table 5.

**Figure 2:**
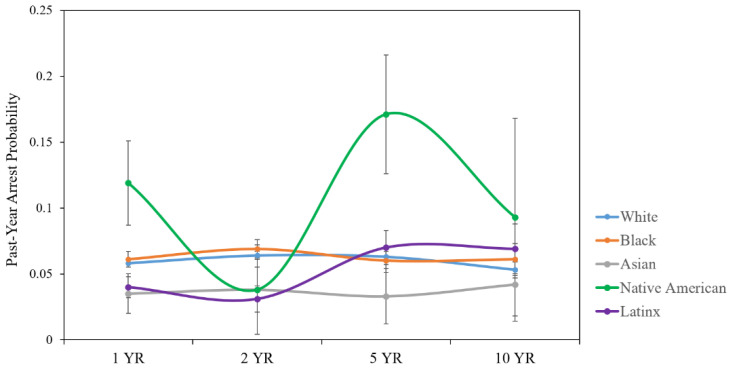
Racial/ethnic differences in past-year arrest probability trajectories over time with covariate-adjusted means and standard error bars.

After adding these covariates into the previously significant models, Native American, Black, and White individuals with TBI still had higher arrest probability trajectories than Asian individuals. Conversely, the differences in arrest probability trajectories previously found between Latinx and Native American, White and Native American, Latinx and Asian, and White and Black individuals were no longer significant after controlling for covariates, though all comparisons except for the White vs. Black comparison were in the marginally significant range (*p* < 0.10). The arrest probabilities for each racial/ethnic group over time with covariate-adjusted means appear in Figure 2.

## 4. Discussion

This study assessed racial/ethnic differences in arrest probability trajectories over the course of ten years after TBI. Arrest probability generally decreased over ten years post-discharge with a slight leveling off or plateauing of the curve, suggesting quadratic movement. Asian individuals with TBI had the lowest overall arrest probability trajectories, followed by the White, Latinx, Black, and then Native American groups. After including demographic and injury-related characteristics as covariates, the racial/ethnic effects remained significant for the Asian vs. Native American, Black vs. Asian, and White vs. Asian models; however, the racial/ethnic effects were no longer significant in the Latinx vs. Native American, White vs. Native American, Latinx vs. Asian, and White vs. Black comparisons, though in most cases the effects approached statistical significance.

The current study hypothesized that Black, Latinx, and Native American individuals with TBI would show higher arrest probability trajectories compared to White and Asian individuals and the results of this study robustly supported this hypothesis. These findings build upon previous literature which conversely did not reveal significant racial/ethnic differences in arrest outcomes post-injury [23,24]. The lack of significant racial/ethnic differences in these previous studies, in contrast to the current study, may have been a function of the creation of orthogonal dummy codes to directly compare all racial/ethnic groups to one another, rather than comparing racial/ethnic minority groups to the White group only. For example, the current study found that Asian individuals with TBI showed the lowest arrest probability trajectories, and as a result any previous studies that lump Asian individuals into a group with other racial/ethnic minorities are likely to have obscured racial/ethnic differences in arrest probabilities.

**Figure 3:**
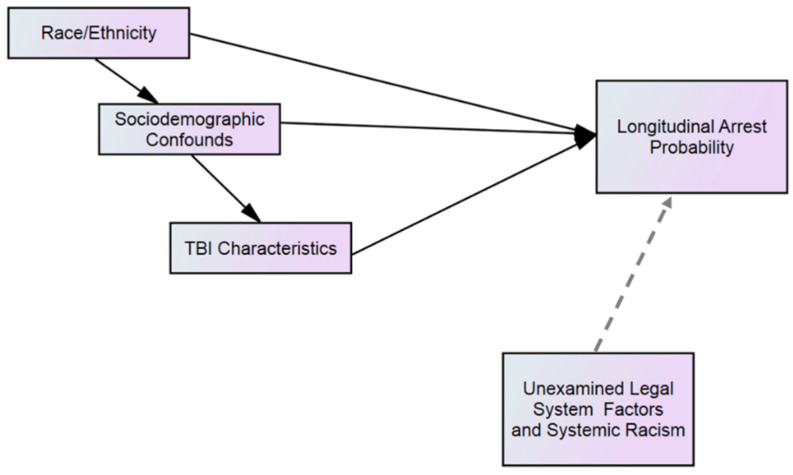
Proposed theoretical model.

The present study findings inform a theoretical framework (Figure 3) outlining that racial/ethnic group membership contributes to differences in sociodemographic variables (e.g., education, employment, etc.), which are then associated with both severity and violent causes of TBI. The model proposes that Black, Latinx, and Native American group membership confers risk for these sociodemographic vulnerabilities, which contribute to TBI characteristic vulnerabilities, which are in turn associated with increased probability of criminal arrests. For example, lower educational achievement and younger age at TBI have been shown to contribute to increased arrest probability among a sample of incarcerated persons [16]. Concerns for arrests within the context of TBI arise in part given the potential effects of cognitive impairment on aggression. Research has shown incarcerated persons with a history of TBI had a higher likelihood of violent and non-violent legal infarction history [28,29]. Although future criminal justice concerns are an important outcome to consider among incarcerated persons with a TBI history, findings in the literature have shown no consistent relationship between aggression or violent criminal offending and TBI [18,30]. Thus, the current model proposes a cascade of sequential effects, but also that each of these variable sets independently confers risk for criminal arrest after TBI, in part because of additional unexamined factors not present in the current study. Importantly, these additional factors can include those from the legal system such as criminal justice policies and policing practices that have been shown disproportionately to target Black, Latinx, and lower income communities [31], as well as systemic racism.

Additionally, many studies that have assessed incarceration rates have failed to include an Asian subsample or categorized this group as “other”. From a criminal justice perspective, Asian individuals are less likely to encounter factors such as harsher sentencing and heavier policing, which are known to contribute to incarceration and arrest outcomes among Black, Latinx, and Native American communities [31,32]. From a TBI perspective, the study showed that Asians with TBI were older, reported fewer substance use issues, experienced higher post-TBI functional independence, and had a lower proportion of severe and violent injuries. The current study findings align with literature showing Black, Latinx, and Native American individuals with TBI to have a higher likelihood of violent injury cause compared to White and Asian groups [5,6,7,8,9,10,11]. Although the current study findings of lower arrest probability trajectories for the Asian subsample may be explained in part by the social inequalities which generally do not specifically target the Asian community in the U.S., most of the racial/ethnic differences in arrest probability trajectories with the Asian subsample remained even after controlling for related demographic confounds.

### 4.1. Clinical and Public Health Implications

The current findings have implications for TBI rehabilitation and public health. A review of the clinical practice guidelines literature conducted by Bayley et al. (2018) generated a series of recommendations for rehabilitation program structure [33]. The guidelines suggested that rehabilitation treatment plans should be goal-oriented, and the application of the present study findings dovetail with this suggestion such that TBI rehabilitation centers should integrate goals that address the malleable predictors of arrest probability, especially among Black, Latinx, and Native American groups. For example, unaddressed preinjury substance use concerns may be a key component for rehabilitation programming. The current study findings underscore the importance of rehabilitation teams aligning individuals with TBI from at-risk backgrounds—particularly those who also are in various combinations of younger age, men, lower education, unemployment at injury, and pre-injury substance use or arrest backgrounds, and with higher functional independence at discharge—with community-based services that emphasize occupational readiness training during treatment by way of employment trials, which could provide a more seamless discharge plan. Given the current findings, it is recommended that rehabilitation clinicians utilize culturally informed support, such as Hay’s ADDRESSING model [34] expressly among these racial/ethnic groups with TBI. For disability-specific interventions, the “Who Am I?” activity [35] facilitates inquiry about intersecting cultural identities that can contribute to wellness within rehabilitation contexts. Such approaches could spur discussions surrounding culture within inpatient or outpatient rehabilitation contexts and lead to the implementation of community-based practices for post-discharge efforts to alleviate racial/ethnic-based risks in arrest probability for people with TBI.

Pre-injury victimization can contribute to arrest outcomes [16], and it is suggested that rehabilitation protocols include the assessment of pre-injury victimization history that could contribute to poorer long-term post-discharge outcomes, especially among the groups that were shown to have higher arrest probability trajectories in the current study (i.e., Native American, Black, and Latinx). The evaluation of injury and functional risks could avail the possibility for additional psychosocial supports during the acute-rehabilitation stage to mitigate arrest likelihood, as well as provide future directions for post-discharge planning. The lower post-TBI arrest rates within veteran and service member populations compared to civilian populations in the previous literature may be in part due to the diverse post-rehabilitation opportunities available to veterans and service members [24]. As such, the current study findings suggest that rehabilitation clinicians aim to connect civilians who sustain TBI with community and post-rehabilitation resources that are akin to those available for veterans and service members who are likely to experience systemic barriers that contribute to arrest outcomes. Unfortunately, not many of these programs exist—and not many payors are willing to reimburse the ones that do. For example, Medicaid rarely pays for physical and occupational therapy in a nursing facility, much less for a transitional or vocational rehabilitation program staffed by an interdisciplinary team in a residential setting. As a result, funding for these types of programs is critical, particularly for individuals with TBI from racial/ethnic minority backgrounds who were not already employed, had a violent cause of injury, or who could not afford to pay for it out of pocket.

### 4.2. Limitations and Future Directions

The current study findings should be interpreted within the context of several limitations, and thus opportunities for future research. Although Native American individuals had the highest trajectories of arrest probabilities, the relatively small subsample of Native Americans (and Asians) may have warped the curves shown in the arrest probability figures; for example, only one Native American person with TBI had been arrested over the past year at the 10-year follow-up data collection. As both Native American and Asian subgroups were notably smaller than the other racial/ethnics groups, this might have biased the results for those groups. Future research should recruit more sizeable samples of Native Americans and Asians with TBI to examine whether this is a true post-TBI arrest trajectory for both populations. Due to the nature of the TBIMS and the current study’s use of the “public” version of the data that omits addresses or geocodes, we were unable to assess these findings with regional difference in mind. TBIMS sites are mostly embedded in urban areas at Level 1 hospitals with quite large catchment areas spanning urban, suburban, and rural regions. As a result, it could be useful for future studies to explore the current research questions across regions and rurality to assess for potential differences and test generalizability of the findings. The current study used cross-sectional data at baseline as predictors; so, it is suggested that future research utilize cross-lagged panel designs or structural equation modeling to identify theoretical causality of racial/ethnic differences in arrest probability, while incorporating unexamined legal system and systemic racism factors. It would also be extremely important to consider predictors of increased arrest probability trajectories within racial/ethnic groups in order to determine which subgroups are at increased risk. These analyses would allow for identification of modifiable factors relevant to the follow-up period.

Mental health treatment history significantly predicted arrest outcomes among veterans [24] and civilian populations [20], but this variable was not available for the current study in sufficient numbers to include. As such, future TBIMS research should incorporate an available assessment of mental health history, and other psychosocial variables to evaluate their role in racial/ethnic differences in arrest probability outcomes. An additional limitation and opportunity for future research is that the current study did not evaluate whether functional changes to the brain were significant predictors of racial/ethnic differences in arrest probability trajectories. Previous research suggests that frontal and parietal lobe damage contributed to higher recidivism rates among individuals with TBI [36,37]; thus, future research should incorporate functional measures of neuroanatomical changes, as well as evaluate differences in these features. Finally, future research should assess the types of daily activities and integration practices that groups who reported lower arrest probability trajectories engage in.

## 5. Conclusions

Results of the current study suggested that racial/ethnic differences in trajectories of arrest probability exist and often remained after covarying for injury and sociodemographic features. These findings suggest that broader unexamined systemic racism factors may better account for arrest probability outcomes among these respective groups. This study supports future exploration of the “acute-rehab to prison pipeline”, especially among Black and Latinx individuals with TBI, and growing evidence for the Native American population. Although the present study showed a rather small proportion of the sample were arrested post-TBI, these data nonetheless underscore profound racial/ethnic differences in arrest outcomes among TBI populations.

## Figures and Tables

**Table 1:** Sample demographic and injury information.

Variable	Omnibus *p*-Value	White (*n* = 9045)	Black (*n* = 2409)	Latinx (*n* = 1316)	Asian (*n* = 344)	Native American (*n* = 81)
Age, *M*	<0.001	42.30 _abc_	37.96 _ade_	36.37 _bd_	41.17 _ef_	37.40 _cf_
Sex, % male	<0.001	71.2 _ab_	77.7 _ac_	77.5 _bd_	70.1 _cd_	69.1
Education, *M*	<0.001	12.98 _abcd_	11.77 _aef_	10.5 _behi_	13.7 _cfgh_	11.99 _dgi_
Competitively Employed, % yes	<0.001	65.1 _abc_	55.7 _ad_	69.3 _bdfg_	60.9 _ef_	47.4 _ceg_
Pre-injury Substance Use, % yes	<0.001	43.0 _ab_	44.4 _cd_	41.3 _fg_	25.8 _acef_	60.5 _bdeg_
Pre-injury arrests, % yes	<0.001	7.2 _abcd_	16.7 _aef_	10.6 _behi_	3.5 _cfgh_	19.8 _dgi_
Injury Cause, % violent	<0.001	6.2 _abcd_	26.4 _aefg_	16.0 _beh_	10.3 _cfh_	14.8 _dg_
Injury Severity, %	0.039	_a_		_ab_	_b_	
Mild		12.6	10.7	10.4	12.9	7.6
Moderate		11.7	11.8	9.4	13.6	13.6
Severe		75.8	77.6	80.2	73.5	78.8
FIM Total at Discharge, *M*	<0.001	91.27 _ab_	88.41 _acd_	89.36 _bef_	93.22 _ce_	96.10 _df_
Past Year Arrests, % yes	<0.001					
Year 1		5.2 _abc_	7.1 _ad_	5.8 _f_	1.6 _bdef_	11.4 _ce_
Year 2		5.6 _ab_	7.7 _acd_	5.4 _df_	2.3 _bcef_	7.6 _e_
Year 5		5.5 _abc_	7.0 _ade_	9.3 _cg_	2.9 _dfg_	16.3 _bef_
Year 10		4.7 _a_	7.5 _a_	6.7	3.9	5.9

*Note*. Values sharing a subscript were significantly different (*p* < 0.05) in follow-up comparisons; all follow-up comparisons were pairwise, except for Injury Severity, %, which for simplicity were holistic across all three severity category levels.

**Table 2:** Correlation matrix.

	1	2	3	4	5	6	7	8	9	10	11	12
1. Sex												
2. Age	−0.091 ***											
3. Education	−0.057 ***	0.139 ***										
4. Employment	0.142 ***	−0.251 ***	0.108 ***									
5. Pre-Injury Substance Use	0.161 ***	−0.271 ***	−0.104 ***	0.053 ***								
6. Pre-Injury Arrest History	0.136 ***	−0.051 ***	−0.180 ***	−0.022 *	0.190 **							
7. Injury Cause	0.104 ***	−0.085 ***	−0.117 ***	−0.039 ***	0.141 **	0.147 ***						
8. Injury Severity	0.113 ***	−0.315 ***	−0.090 ***	0.143 ***	0.128 ***	0.059 ***	0.020 *					
9. FIM	0.043 ***	−0.121 ***	0.062 ***	0.118 ***	0.090 ***	0.015	0.028 **	−0.075 ***				
10. Arrests: Year 1	0.078 ***	−0.133 ***	−0.093 ***	−0.019 *	0.152 ***	0.118 ***	0.050 ***	0.057 ***	0.086 ***			
11. Arrests: Year 2	0.078 ***	−0.141 ***	−0.098 ***	−0.003	0.150 ***	0.105 ***	0.065 ***	0.038 ***	0.088 ***	0.260 ***		
12. Arrests: Year 5	0.078 ***	−0.138 ***	−0.106 ***	−0.026 *	0.136 ***	0.119 ***	0.033 **	0.048 ***	0.077 ***	0.162 ***	0.194 ***	
13. Arrests: Year 10	0.068 ***	−0.125 ***	−0.090 ***	−0.030	0.103 **	0.081 ***	0.043 **	0.022	0.044 **	0.073 ***	0.108 ***	0.145 ***

Note. Values represent correlation coefficients. * = *p* < 0.05, ** = *p* < 0.01, *** = *p* < 0.001.

**Table 3:** Main effect of race/ethnicity on arrest probability trajectory.

Comparison	*b*-Weight	*p*-Value	95% CI
White vs.			
Black	−0.02	<0.001	−0.03, −0.01
Latinx	−0.01	0.059	−0.02, 0.00
Asian	0.03	<0.001	0.01, 0.05
Native American	−0.06	0.002	−0.09, −0.02
Black vs.			
Latinx	0.01	0.117	−0.00, 0.02
Asian	0.05	<0.001	0.03, 0.07
Native American	−0.04	0.093	−0.08, −0.00
Latinx vs.			
Asian	0.04	<0.001	0.02, 0.06
Native American	−0.05	0.028	−0.09, −0.01
Asian vs.			
Native American	−0.09	<0.001	−0.12, −0.05

**Table 4:** Examining differential change in arrest probability trajectories over time as a function of race/ethnicity.

Interaction with Quadratic Time	*b*-Weight	*p*-Value	95% CI
White vs. Black * Time^2^	−0.0003	0.416	−0.001, 0.0004
White vs. Asian * Time^2^	−0.0002	0.861	−0.002, 0.002
White vs. Native American * Time^2^	0.002	0.252	−0.002, 0.006
Black vs. Asian * Time^2^	0.0002	0.890	−0.002, 0.002
Latinx vs. Asian * Time^2^	−0.001	0.330	−0.003, 0.001
Latinx vs. Native American * Time^2^	0.001	0.537	−0.003, 0.006
Asian vs. Native American * Time^2^	0.002	0.196	−0.001, 0.006

**Table 5:** Main effect of race/ethnicity on arrest probability trajectory controlling for covariates.

Comparison	*b*-Weight	*p*-Value	95% CI
White vs.			
Black	−0.00	0.900	−0.01, 0.01
Asian	0.02	0.034	0.00, 0.04
Native American	−0.04	0.073	−0.08, 0.00
Black vs.			
Asian	0.04	0.008	0.01, 0.06
Latinx vs.			
Asian	0.02	0.055	−0.00, 0.05
Native American	−0.05	0.062	−0.09, 0.00
Asian vs.			
Native American	−0.08	<0.001	−0.13, −0.04

## Data Availability

Data are available upon request made to the corresponding author.
